# Structural brain alterations in patients with anxious depression: evidence from the REST-meta-MDD project

**DOI:** 10.3389/fpsyt.2025.1589040

**Published:** 2025-07-25

**Authors:** Songhao Hu, Li Zhu, Xiang-Yang Zhang

**Affiliations:** ^1^ Department of Child and Adolescent Psychiatry, Hefei Fourth People’s Hospital, Hefei, Anhui, China; ^2^ School of Mental Health and Psychological Sciences, Anhui Medical University, Hefei, Anhui, China

**Keywords:** major depressive disorder, anxious depression, gray matter volume, structural covariance, support vector regression

## Abstract

**Background:**

Anxious depression (AD) is a clinically significant subtype of major depressive disorder (MDD) characterized by prominent anxiety symptoms. Emerging neuroimaging evidence shows that AD patients have significantly altered brain structure. This study aimed to identify reliable neuroimaging biomarkers for AD in a Chinese cohort.

**Methods:**

Participants were recruited from the REST-meta-MDD project, including 178 MDD patients and 89 healthy controls. MDD patients were stratified into 89 patients with AD and 89 with non-anxious depression (NAD). Voxel-based morphometry (VBM) was used to quantify gray matter volume (GMV) using T1-weighted images. Depressive and anxiety symptoms were assessed using the Hamilton Depression Rating Scale (HAMD-17) and the Hamilton Anxiety Rating Scale (HAMA-14). Structural covariance (SC) analysis was employed to investigate coordinated morphological changes across brain regions. Additionally, a support vector regression (SVR) model was constructed to predict anxiety severity in MDD patients, with external validation performed in an independent dataset.

**Results:**

In AD patients, significant increases in GMV were observed in the right precuneus (PCUN) and right superior parietal gyrus (SPG). Reduced SC was also found between the right PCUN and left anterior cingulate gyrus (ACG), as well as between the right PCUN and right angular gyrus (ANG). Additionally, SVR analysis demonstrated that the right PCUN GMV could effectively predict MDD patients’ HAMA-14 scores (*r* = 0.477, MSE = 73.865), validated in an independent external dataset (r = 0.368, MSE = 100.961).

**Conclusions:**

This study’s findings indicate that brain structural abnormalities may be a crucial pathophysiological basis for AD.

## 1 Introduction

Major depressive disorder (MDD) represents a complex global mental disorder characterized by persistent depressive mood and anhedonia, with a global prevalence of approximately 185 million people (1, 2). According to a 2013 epidemiological study, MDD has become the leading cause of disability in China, imposing a substantial burden on both individual functioning and public health systems (3). MDD exhibits substantial heterogeneity in terms of clinical presentations, pathogenic mechanisms, and treatment responses, which has led to an increasing recognition that it may represent a spectrum of disorders rather than a single disease entity (4, 5). The challenges of accurately diagnosing and effectively treating MDD make it imperative to focus on understanding its different subtypes, thus facilitating the advancement of more personalized and effective therapeutic approaches (4).

Among the various clinical presentations of MDD, anxious depression (AD) has emerged as a predominant subtype, manifesting prominent anxiety features and occurring in roughly 45.7% of MDD patients (6). Clinical investigations have demonstrated that AD patients present distinct clinical profiles when contrasted with their non-anxious depression (NAD) counterparts, including exacerbated symptom severity, pronounced functional impairment, elevated relapse susceptibility, diminished therapeutic responsiveness, and increased suicidality risk (6–10). The differential characteristics between AD and NAD have been extensively examined across multiple domains, encompassing neurobiological mechanisms, phenotypic expressions, and molecular biomarkers (11, 12).

The field of neuroimaging has made significant strides in elucidating the neural correlates of anxiety disorders (AD), with resting-state magnetic resonance imaging (rs-MRI) emerging as a pivotal tool for identifying depression-related neurobiological subtypes (4, 13, 14). Among these, structural magnetic resonance imaging (sMRI) studies have consistently revealed distinct patterns of neuroanatomical alterations in AD patients. Emerging evidence from a recent brain network study has established a significant association between the presence of anxiety symptoms and reduced cortical volumes in key regions of the default mode network (DMN) in affected patients (15). Voxel-based morphometry (VBM) investigations have additionally uncovered notable decreases in grey matter volume (GMV) within the frontal and temporal lobes of AD patients (16, 17). Moreover, Zhou et al. effectively employed a random forest classification model using multimodal MRI features, achieving a high classification accuracy in identifying AD patients (AUC = 0.802) (18). Therefore, investigating neuroimaging biomarkers for AD based on sMRI data represents a feasible and promising strategy, offering potential avenues for advancing diagnostic accuracy and personalized therapeutic interventions in future research.

However, most prior studies have focused solely on structural changes in isolated brain regions in AD, overlooking the disruption of structural association, a key feature of its multiregional collaborative pathology. Structural covariance (SC), a well-established neuroimaging methodology based on sMRI data, provides a reliable characterization of coordinated morphological variations across cerebral cortical regions (19). It provides partial insight into the interregional connectivity patterns within the brain. Compared to functional connectivity (FC), which examines inter-regional associations based on functional similarity derived from fMRI data, SC demonstrates more stable connectivity features (20, 21). Previous studies by Chen et al. have demonstrated that patients with anxiety disorders exhibit distinct structural connectivity patterns between the anterior cingulate cortex (ACC) and prefrontal cortex (PFC) (22). Therefore, exploring the pattern of SC changes in AD patients based on GMV alterations that characterize AD patients holds significant potential for elucidating the underlying neuropathological mechanisms of AD.

Moreover, machine learning has emerged as a novel analytical approach in recent years, offering powerful tools to elucidate the underlying mechanisms linking neuroimaging alterations with core clinical manifestations of mood disorders (23). Support Vector Regression (SVR) has been widely adopted in sMRI analyses due to its superior performance in modeling the relationship between pathological changes and clinical symptoms, making it a well-established machine learning approach in the field (24, 25). Consequently, this study employs SVR to examine the relationship between GMV in specific brain regions and anxiety levels.

As far as we are aware, no studies have yet investigated structural brain alterations in Chinese AD patients by combining VBM and SC methods. In this study, we first identified GMV alterations in AD patients. Subsequently, we integrated SC analysis based on these GMV changes to investigate structural covariance patterns among brain regions. Finally, the clinical relevance of the identified regions was further validated using an SVR model. Based on the available evidence, we proposed three main hypotheses: 1) AD patients would exhibit unique patterns of structural brain alterations; 2) AD patients exhibit distinct patterns of SC alterations across brain regions; and 3) these specific structural alterations would predict the severity of anxiety symptoms in MDD patients.

## 2 Materials and methods

### 2.1 Participants

The study participants were derived from the REST-meta-MDD project, encompassing 25 research cohorts across 18 Chinese medical institutions (26, 27). A comprehensive demographic and clinical profile was established through systematic collection of key variables, including diagnostic status, sociodemographic characteristics (age, gender, educational attainment), and psychometric assessments using the 14-item Hamilton Anxiety Rating Scale (HAMA-14) and 17-item Hamilton Depression Rating Scale (HAMD-17). However, the lack of detailed documentation regarding medication protocols and disease progression in this project unfortunately precludes a comprehensive evaluation of their potential impact on the study outcomes.

Before being enrolled in the study, all participants gave written informed consent, which had been sanctioned by institutional review boards. The research protocol received ethical clearance from local Institutional Review Boards, with subsequent data sharing authorization granted by the Ethics Committee of the Institute of Psychology, Chinese Academy of Sciences, following the complete deidentification of participant information. These rigorous ethical safeguards were implemented to maintain research integrity and protect participant rights throughout the study.

Thise study recruited MDD participants who satisfied the inclusion criteria: 1) aged at least 15 years; 2) a confirmed diagnosis of MDD through a Structured Clinical Interview based on the Diagnostic and Statistical Manual of Mental Disorders-IV (DSM-IV) or International Classification of Diseases 10 (ICD-10); and 3) a HAMD-17 total score ≥17 at the baseline assessment before neuroimaging. Additionally, 88 individuals diagnosed with MDD and 38 healthy controls were excluded from the analysis (28). This study implemented rigorous exclusion criteria to ensure data quality and homogeneity: 1) exclusion of patients with late-onset depression or patients in remission; 2) exclusion of participants missing basic demographic data (gender, age, or education level); 3) exclusion of imaging data that did not meet the quality control criteria, particularly those with inadequate spatial normalization.

Based on the aforementioned criteria, we enrolled a total of 178 patients with MDD and 89 healthy controls as the primary dataset for subsequent analyses (see in **Supplementary Table 1**). Additionally, an independent external validation dataset comprising 20 MDD patients and 20 healthy controls was included to evaluate the generalizability of the SVR model. The external validation dataset was selected based on the following inclusion criteria: 1) participants aged 15 years or older, and 2) a confirmed diagnosis of MDD established through DSM-IV or ICD-10. To ensure data quality and homogeneity, stringent exclusion criteria were applied: 1) patients with late-onset depression or those in remission were excluded; 2) participants lacking essential demographic data (gender, age, or education level) were removed; 3) imaging data failing to meet quality control standards—particularly those with inadequate spatial normalization—were discarded.

### 2.2 Clinical measures

In this study, two well-validated psychometric instruments were used for symptom assessment: the HAMD-17 for quantifying depression severity and the HAMA-14 for assessing anxiety severity in MDD patients. Both instruments have demonstrated reliable psychometric properties in the Chinese population, with scale scores positively correlating with symptom severity; higher HAMD-17 scores indicate more pronounced depressive features, while the higher HAMA-14 scores reflect greater anxiety intensity (29, 30).

In the primary dataset, AD classification was determined through HAMD-17 and HAMA-14. MDD patients meeting the criteria of HAMD-17 scores ≥17 and HAMA-14 scores ≥14 were classified into the AD group, while those with HAMD-17 scores ≥17 but HAMA-14 scores<14 were classified into the anxious depression (NAD) group (31). This classification scheme has been empirically validated in prior research (31–34). In addition, we selected 89 healthy subjects as the healthy controls (HC) group; none of the HC group had anxiety or depression symptoms. Notably, due to the absence of HAMD scale data in the external validation dataset, MDD patients with HAMA-14 scores ≥14 were classified into the AD group, while the remaining MDD patients were designated as the NAD group.

### 2.3 MRI data acquisition, preprocessing, and quality control

In this research, T1-weighted structural MRI images were processed utilizing the DPARSF software for initial preparation (35). The image analysis was conducted utilizing the SPM 8 software in conjunction with the VBM 8 toolbox (http://dbm.neuro.unijena.de/vb) (26). T1 images were normalized using template space and subsequently segmented into grey matter (GM), white matter (WM), and cerebrospinal fluid (CSF). To align the individual grey matter and white matter images to MNI space, we applied the normalization function from the Diffeomorphic Anatomical Registration Through Exponentiated Lie Algebra (DARTEL) toolbox. Finally, the grey matter maps of each subject were smoothed with an 8 mm full-width at half-maximum (FWHM) Gaussian kernel.

### 2.4 Analysis of SC

In this study, we adopted a data-driven approach, selecting seed regions exhibiting significant between-group differences in GMV to perform SC analysis (19, 20). By examining the relationships between these seed regions and whole-brain voxel-wise GMV, we investigated distinct SC alteration patterns in AD patients. Furthermore, to compare SC differences between AD and NAD groups, we constructed an interaction linear model.


$$T_{i}=\beta 0+\beta 1Tj+\beta 2Group+\beta 3(Tj\times Group)+\beta 4Tn+\in$$

To examine differences in GMV correlation slopes between seed areas and other brain regions across groups (AD *vs*. NAD), the interaction model was employed. Here, Ti indicated the average GMV of a seed region, while *Tj* represented voxel-wise GMV values across the brain. The two groups were assigned to the same variable in the model (Group), allowing the model to capture group-specific effects (AD *vs*. NAD) on the slope of GMV correlations between seed regions and other brain voxels. Coefficients *β*0 to *β*4 were estimated, where β0 denoted the intercept, *β*1 captured the *Ti*-*Tj* association, *β*2 reflected group effects, and *β*3 quantified the effect of the interaction. In addition, *β*4 and *Tn* indicate the removal of the effect of covariates (e.g., gender, age, education, site factor). Regression slope differences between groups were evaluated using Student’s *t*-tests applied to the interaction terms. The resulting statistical maps were corrected for multiple comparisons using false discovery rate (FDR) Multi-comparison correction, revealing significant clusters showing between-group differences in SC.

### 2.5 SVR analysis and independent external validation

To evaluate the predictive capacity of GMV alterations in MDD patients for anxiety severity, we constructed a support vector regression (SVR) model using the LIBSVM toolbox (http://www.csie.ntu.edu.tw/~cjlin/libsvm/) (36). The model incorporated GMV values from all voxels within regions showing significant differences among the three groups as initial features. We employed leave-one-out cross-validation (LOOCV) for dataset partitioning and model evaluation (37). Feature selection was performed by retaining only those voxels whose GMV values showed statistically significant correlations with HAMA scores (*p*< 0.05) in each LOOCV iteration. The SVR model was implemented with parameter C set to 10 while maintaining default values for other parameters. Predictive accuracy was quantified by calculating the Pearson correlation coefficient between the model’s predicted values and actual clinical scores. The statistical significance of prediction results was verified through 1000 permutation tests. Finally, we identified “consensus features” as those voxels consistently selected across all LOOCV iterations (38). The weight coefficients of these consensus features were mapped onto brain templates for spatial visualization.

Furthermore, we employed an independent external validation cohort (comprising 20 AD and 20 NAD patients) to assess the model’s performance. Using the consensus features, we reconstructed the model with the original parameters and rigorously evaluated its efficacy in external validation.

### 2.6 Statistical analysis

Statistical analyses were conducted with SPSS 25.0 (IBM Corp., Armonk, NY). Intergroup differences among the three groups were analyzed using one-way analysis of variance (ANOVA) for continuous variables (age and years of education) and chi-square tests for categorical variables (gender distribution). Clinical characteristics, including HAMD-17 and HAMA-14 scores, were compared between AD and NAD groups using independent samples *t*-tests. The statistical significance threshold was maintained at *p*< 0.05 for all analyses.

To examine GMV variations across the three groups (AD, NAD, and HC), we conducted comprehensive whole-brain analyses employing ANCOVA. Additionally, to control for potential confounding effects and account for site-related variability, we included sex, age, educational level, and site effects as covariates in the statistical models, following established practices in prior research (27, 39). Between-group comparisons were subsequently performed using *post hoc t*-tests with multiple comparison corrections, implementing a dual-threshold approach (voxel-level *p*< 0.001 combined with cluster-level FDR correction at *p*< 0.05). Given the significant age differences among the three groups in this study, we conducted a sensitivity analysis with age stratification to enhance the robustness of our findings. Based on previous studies (40, 41), the primary dataset was stratified into three age-based subgroups:<25 years (young group), 25–40 years (core adulthood group), and >40 years (late adulthood group). Subsequent intergroup analyses were conducted within these subgroups, comparing AD, NAD, and HC groups.

Furthermore, region-specific GMV values from significantly different clusters were extracted for subsequent correlation analyses with clinical measures of anxiety and depression severity, while controlling for demographic variables (age, sex, educational attainment, and site effects).

## 3 Results

### 3.1 Demographic data between the groups

In the primary dataset, demographic characteristics were similar across the three groups, with the only significant difference observed in age (*p*< 0.05). The AD group showed elevated anxiety levels on HAMA-14 compared to NAD controls (*p*< 0.05), while depressive symptoms measured by HAMD-17 remained comparable between groups (*p* > 0.05) (**Table 1**).

**Table 1:** Demographic and clinical characteristics of the primary dataset.

Characteristics (mean±SD)	MDD		HC(n = 89)	Statistical value	*p*-value
	AD (n = 89)	NAD (n = 89)			
Gender (M/F) ^a^	28/61	40/49	40/49	4.48	0.107
Age (years) ^b^	39.93±14.76	29.93±10.66	31.44±11.34	16.87	0.001*
Education (years) ^b^	11.25±3.55	11.75±3.26	13.80±3.51	0.99	0.989
HAMD-17 ^c^	22.08±3.39	21.45±4.19	NA	1.10	0.272
HAMA-14 ^c^	25.36±6.79	9.74±3.18	NA	19.66	0.001*

HC, healthy controls; MDD, major depressive disorder; AD, anxious MDD; NAD, non-anxious MDD.

aChi-square test.

bOne-way ANOVA.

c*t*-tests.

*p< 0.05.

In the external validation dataset, no significant differences were observed in demographic characteristics between the AD and NAD groups, except for HAMA-14 scores (**Table 2** **).**

**Table 2:** Demographic and clinical characteristics of the external validation dataset.

Characteristics (mean±SD)	AD (n = 20)	NAD (n = 20)	Statistical value	*p*-value
Gender (M/F) ^a^	8/12	11/9	1.200	0.342
Age (years) ^b^	26.60±8.04	29.55±7.64	1.190	0.241
Education (years) ^b^	10.35±5.09	9.70±6.91	0.339	0.737
HAMA-14 ^b^	19.90±8.77	9.20±2.97	5.168	< 0.001*

AD, anxious MDD; NAD, non-anxious MDD.

aChi-square test.

b*t*-tests.

*p< 0.05.

### 3.2 Brain regional differences in GMV between AD, NAD, and HC groups

**Figure 1:**
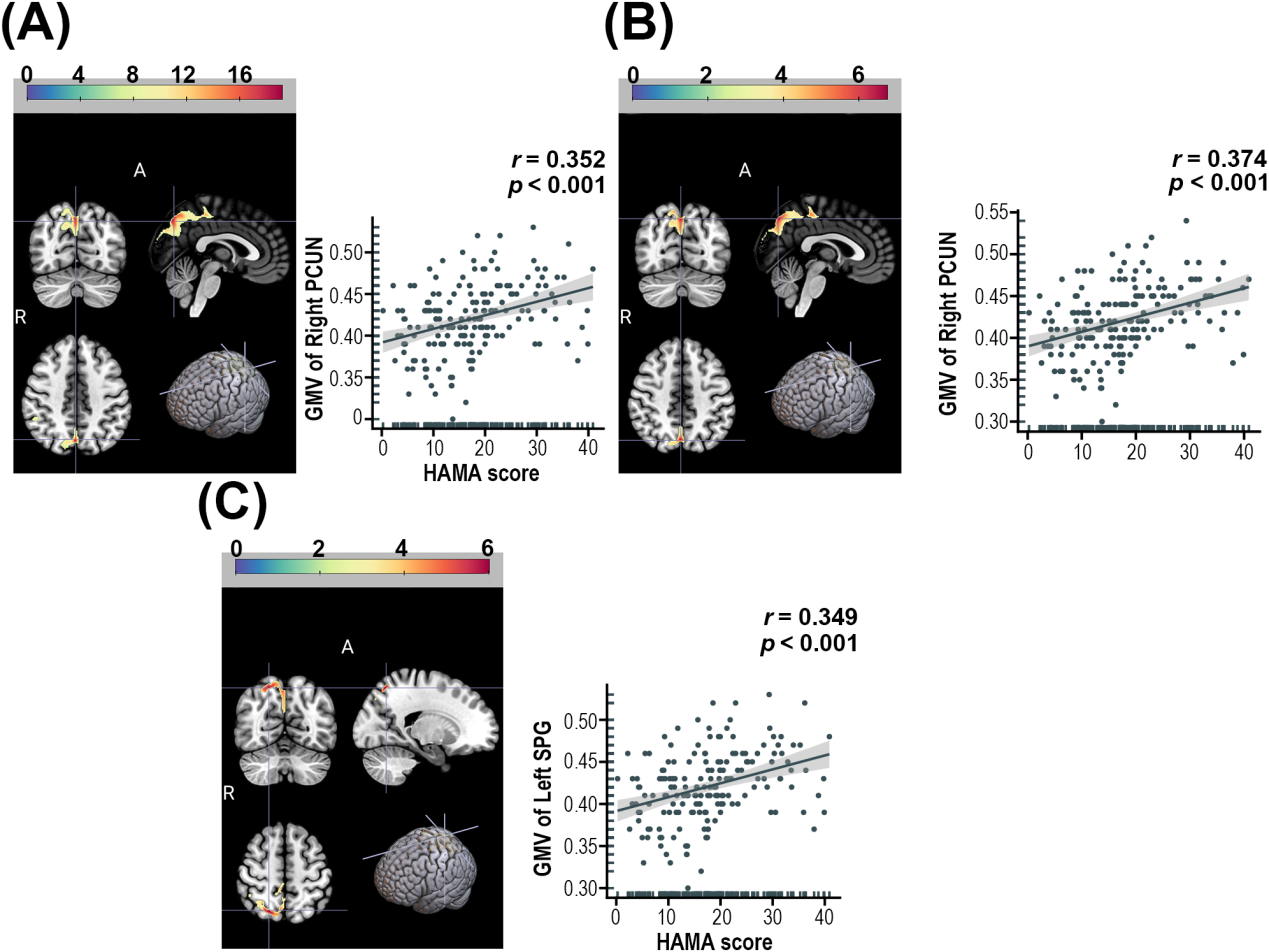
**(A)** Brain regions showed significant differences in GMV among the AD, NAD, and HC groups, and their correlations with HAMA-14 scores. **(B)** Brain regions with significant GMV differences between AD and NAD groups, and their correlations with HAMA-14 scores. **(C)** Brain regions demonstrating significant GMV differences between AD and HC groups, and their correlations with HAMA-14 scores. PCUN, Precuneus; SPG, Superior parietal gyrus. The color bars indicate the *t*-value or *F*-value (voxel-*p*< 0.001, cluster-*p<* 0.05, FDR correction).

ANOVA revealed significant regional differences of GMV in the right precuneus (PCUN) among the AD, NAD, and HC groups (**Table 3**, **Figure 1A**). *Post-hoc* analysis revealed that the AD group exhibited significantly higher GMV in the right PCUN compared to the NAD group (**Table 3**, **Figure 1B**). Furthermore, relative to the HC group, the AD group demonstrated increased GMV in the SPG (**Table 3**, **Figure 1C**).

**Table 3:** Brain areas with significantly different GMV among AD, NAD, and HC groups.

Brain areas	Cluster size (voxels)	MNI coordinates			Peak *F*/*t* value
		X	Y	Z	
Three groups					
Right PCUN	3120	1.5	-70.5	48	19.757
AD > NAD					
Right PCUN	2031	1.5	-70.5	48	6.949
AD > HC					
Right SPG	3004	19.5	-64.5	55.5	6.133

GMV, gray matter volume; PCUN, Precuneus; SPG, Superior parietal gyrus.

Moreover, given the observed age distribution differences among the three groups in this study, we conducted additional sensitivity analyses by stratifying participants into three age subgroups. Replicating the primary analyses within these subgroups consistently revealed brain regions with significant differences among the three groups, further supporting our findings (see **Supplementary Figures 1**–**3**).

After controlling for sex, age, education level, and site information, the correlation analysis in all MDD patients in this study demonstrated that the total HAMA-14 score was significantly associated with the three brain regions showing significant differences in the above analysis (*p*< 0.05) (**Figure 1**).

### 3.3 Differences in SC among AD, NAD, and HC groups

**Figure 2:**
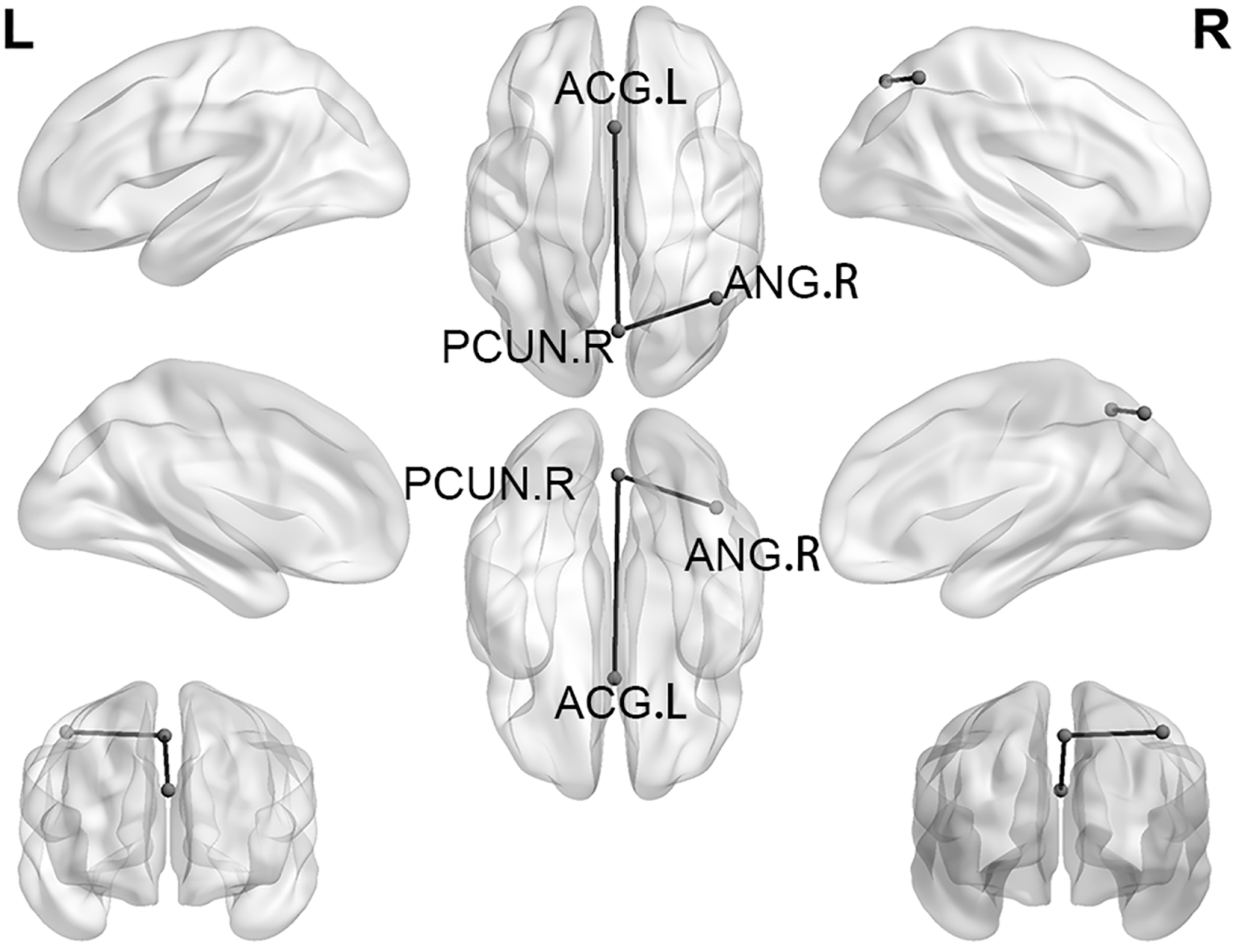
SC with significant differences between the AD group and the NAD group. SC, Structural covariance; PCUN, Precuneus; ACG, Anterior cingulate and paracingulate gyri; ANG, Angular gyrus.

Based on the significant between-group differences identified by ANOVA, we conducted SC analysis. As illustrated in **Figure 2** and detailed in **Table 4**, the AD group exhibited significantly reduced SC between the right PCUN and left anterior cingulate gyrus (ACG), as well as between the right PCUN and the right angular gyrus (ANG), compared to the NAD group.

**Table 4:** Brain areas with significantly lower SC in AD than in NAD groups.

Seed brain area	Brain areas	Cluster size (voxels)	MNI coordinates			Peak *t* value
			X	Y	Z	
Right PCUN	Left ACG	15	0	13.5	25.5	4.292
	Right ANG	184	42	-57	49.5	5.774

SC, Structural covariance; PCUN, Precuneus; ACG, Anterior cingulate and paracingulate gyri; ANG, Angular gyrus.

### 3.4 SVR prediction results

**Figure 3:**
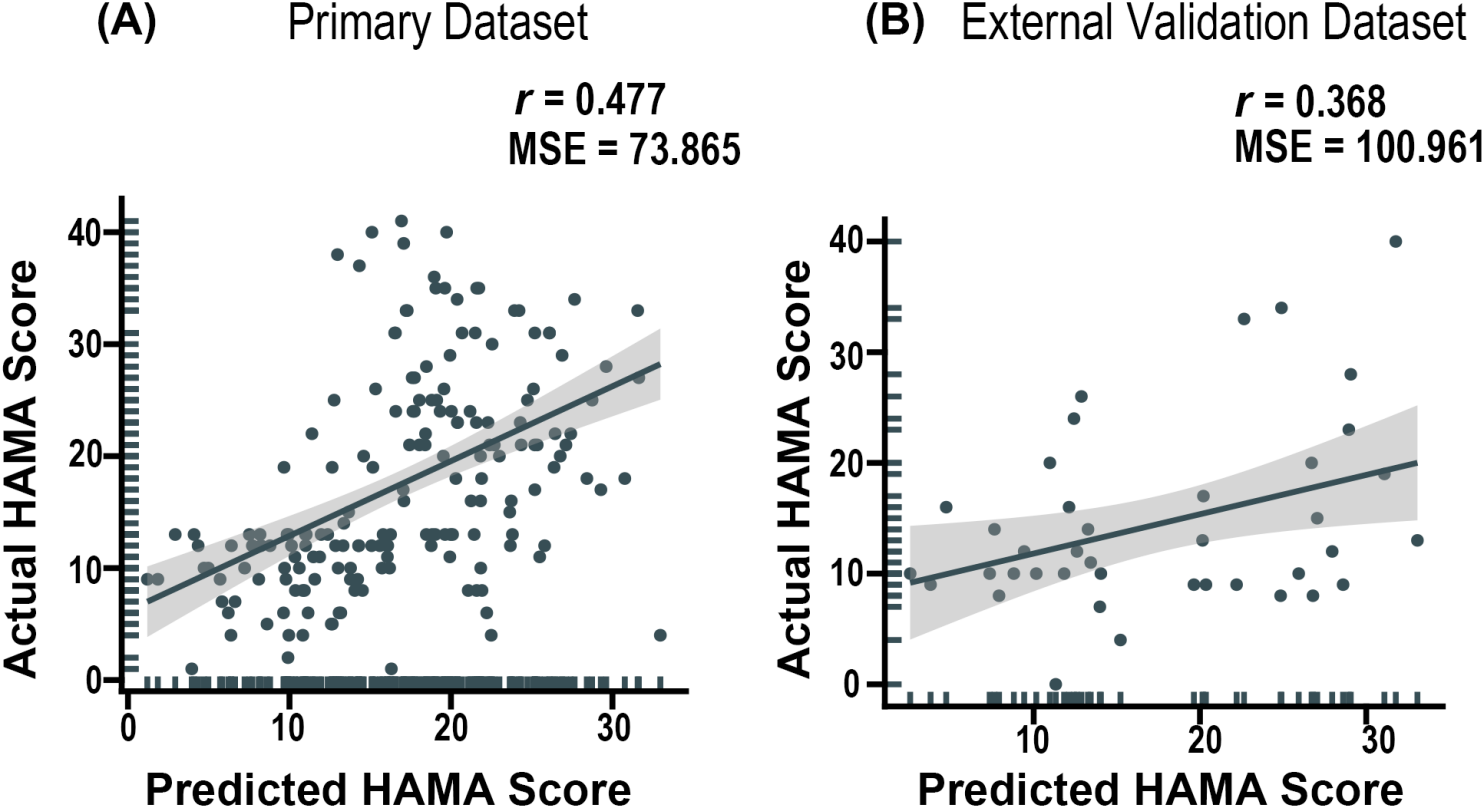
Predictive efficacy of a support vector regression model based on the right PCUN-derived GMV for HAMA Scores. **(A)** Performance Evaluation in the primary datasets. **(B)** Performance Evaluation in the external validation datasets. PCUN = Precuneus.

In this study, we employed an SVR model constructed using all voxels within brain regions exhibiting significant between-group differences in the ANOVA analysis to predict the anxiety severity in MDD patients. Regression analysis revealed that the predicted HAMA-17 scores derived from the SVR model in the primary dataset showed a significant positive correlation with the actual HAMA-17 scores (*r* = 0.477, MSE = 73.865), with the significance confirmed by 1000 iterations of permutation testing (*p*< 0.001) (see **Figure 3**).

**Figure 4:**
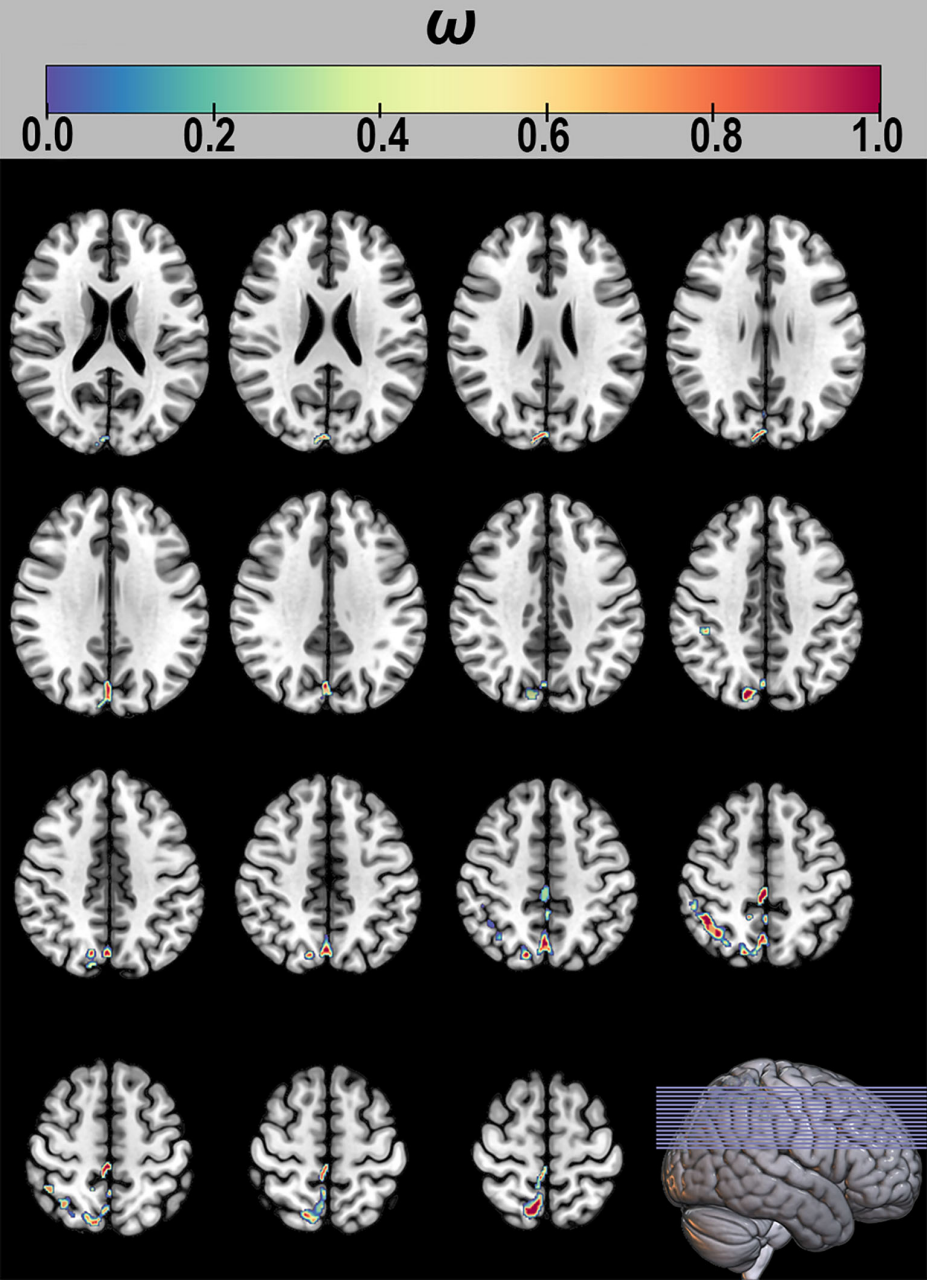
Consensus features and their weight distribution in the support vector regression Model.

Furthermore, in the interpretability analysis, the SVR model identified voxels consistently selected across all LOOCV iterations as ‘consensus features’, and their corresponding weights were examined. The results demonstrated that GMV in five key regions—the inferior parietal lobule (IPL), cuneus (CUN), supramarginal gyrus (SMG), and PCUN—contributed substantially to the model’s predictive performance (see **Figure 4**).

### 3.5 External validation results of the SVR model

Furthermore, the SVR model was externally validated using an independent dataset, demonstrating consistently excellent performance (*r* = 0.368, MSE = 100.961) (see **Figure 3**).

## 4 Discussion

This study investigated the clinical and structural brain characteristics of AD patients using a multicenter MRI dataset. Three key findings were identified (1): Significant differences in GMV were observed among the three groups in the right PCUN. Specifically, the AD group exhibited significantly increased GMV in the right PCUN compared to the NAD group. Furthermore, relative to the HC group, the AD group showed elevated GMV in the right SPG. And these regional GMV alterations were significantly correlated with anxiety severity scores (2). We observed significantly diminished SC in AD patients, particularly between the right PCUN and both the left ACG and the right ANG (3). Leveraging structural changes in AD patients, we established an SVR model with superior predictive accuracy for evaluating anxiety severity in MDD patients, which was robustly validated in an independent external dataset.

In this study, we observed significant differences in the PCUN among the AD, NAD, and HC groups. *Post hoc* analysis revealed that compared to the NAD group, the AD group exhibited abnormally increased GMV in the PCUN region. Furthermore, we identified a significant correlation between this region and anxiety level scores, further underscoring the unique relevance of the PCUN in AD. The PCUN not only plays a critical role in the DMN during resting-state brain activity, but also serves as a central component of the theory of mind (ToM) neural circuitry (42, 43). ToM refers to the capacity to understand and predict the mental states of others based on their actions or experiences (44). Increased GMV in the PCUN may enhance the functioning of ToM neural circuitry, promoting excessive self-referential processing and heightened sensitivity to the perceptions of others, which can contribute to anxiety-related symptoms. Zhou et al. found that patients with AD exhibited higher regional homogeneity (ReHo) in the PCUN, suggesting enhanced functional activity in this region in AD patients. This finding further supports our results, suggesting that structural and functional alterations in the PCUN may contribute to the exacerbation of anxiety symptoms in patients with MDD. These findings suggest that the PCUN may serve as a potential neuroimaging biomarker for AD. In addition, this study revealed that the GMV of the SPG was significantly larger in the AD group compared to the HC group. As a key component of the dorsal attentional network (DAN), the SPG plays a critical role in top-down attentional control, memory retrieval, and spatial task processing (45–47). Recent studies have further demonstrated that heightened functional connectivity within certain regions of the DAN, including the parietal lobe, is significantly associated with anxiety scores (48). The underlying mechanism may involve impaired emotion regulation due to disruptions in DAN-related brain regions, which could contribute to the development of anxiety symptoms (49). Supporting this notion, a recent study reported elevated ReHo in the SPG of AD patients (18). These findings collectively suggest that structural and functional abnormalities in the SPG of AD patients may disrupt emotion regulation, thereby promoting the emergence of anxiety symptoms. Moreover, the emotional and cognitive symptoms associated with SPG dysfunction may exacerbate these disruptions in emotion regulation, potentially leading to a vicious cycle. In this cycle, impaired emotion regulation could further aggravate cognitive decline and behavioral symptoms such as anxiety and depression (49). Future research should explore whether targeted interventions, such as transcranial magnetic stimulation (TMS) therapy focusing on the SPG region and cognitive therapy for anxiety symptoms, can alleviate anxiety symptoms and enhance overall cognitive functioning in AD patients. These interventions may provide a promising approach to addressing the complex interplay between structural abnormalities and neuropsychiatric symptoms in AD. Anxiety symptoms are prevalent in patients with MDD and can contribute to the development of AD, which is characterized by heightened agitation and restlessness (50, 51). The comorbidity of anxiety and depression can exacerbate disease progression and elevate the risk of suicide in MDD patients (7, 10, 50–52).

In this study, we selected the right PCUN region, which demonstrated significant differences in the three-group ANCOVA analysis, as a seed region for voxel-wise SC analysis. Our findings revealed that compared to the NAD group, the AD group exhibited significantly reduced SC between the right PCUN and both the left ACG and right ANG. This suggests that AD patients display a distinct pattern of structural connectivity alterations compared to NAD patients. In the present study, we identified significant SC-induced alterations in the PCUN, ACG, and ANG, all of which are key components of the DMN (53, 54). The DMN is implicated in self-referential thought and emotional control, with evidence suggesting its critical involvement in impaired affective processing observed in depression (55, 56). Research indicates that the functional connectivity within the DMN reflects sustained self-referential processing in the absence of external stimuli and is associated with maladaptive rumination (57). This psychological mechanism may lead individuals with MDD to exhibit excessive focus on their emotional states and negative life events (58, 59). Such maladaptive rumination on negative attributes potentially exacerbates anxiety symptoms, thereby contributing to the development of AD. Moreover, the ACG, a key hub of the DMN, also serves as a critical component of the ACC. Notably, ACC dysfunction has been widely recognized as a hallmark feature of anxiety disorders (60). Lueken et al. reported in their investigation that ACC activity may serve as a predictive biomarker for treatment response in anxiety disorders (61). Existing studies have demonstrated that weakened functional connectivity between the ACC and other brain regions might represent a neural signature of anxiety, which aligns consistently with the findings of our current study (62). In AD, the ACC contributes to both affective regulation and executive functions. Its interaction with the amygdala likely facilitates the evaluation of emotional stimuli and the subsequent initiation of appropriate behavioral responses (63). The amygdala may further modulate top-down emotional regulation through its connections with the medial prefrontal cortex (mPFC). A recent study investigating the amygdala in AD patients further substantiates this hypothesis (51). Therefore, we propose that in patients with AD, the ACC may contribute to anxiety symptoms through its role in emotional regulation and executive functions, as well as via its relation with the amygdala. This perspective has also been confirmed in studies specific to anxiety disorders (64, 65). This may represent the neuropathological mechanism underlying anxiety symptoms in AD patients in the present study.

Currently, the assessment of anxiety severity in patients with MDD depends heavily on subjective patient reports and clinicians’ empirical assessments, highlighting the urgent need for objective MRI-based methodologies to quantify anxiety levels in this patient population (18). In this study, we extracted the GMV of all voxels in the right PCUN showing significant intergroup differences and used these features to construct an SVR model. Our results demonstrated that the GMV of the right PCUN has predictive value for HAMA scores in individuals with major depressive disorder (MDD). This finding was further validated in an independent external dataset. Furthermore, in the interpretability analysis of the SVR model, we identified the five brain regions with the highest contribution weights, including the IPL, CUN, SMG, and PCUN. The results demonstrated that these features could predict the HAMA-14 scores to some extent. Similar studies have been conducted previously. For instance, Zhou et al. used multimodal MRI data to develop a diagnostic predictive random forest model that included GMV, low-frequency fluctuation (fALFF), ReHo, and functional connectivity values in the brain regions showing significant differences among AD, NAD, and HC groups (18). Their model achieved a notable classification performance with an AUC of 0.802. Comparing these two studies, we observed that the features used for predictive modelling overlapped, particularly in the PCUN regions. This suggests that MRI-based features, especially in the PCUN regions, are feasible for constructing predictive models of anxiety symptoms. In light of these findings, subsequent studies should focus on the development of standardized MRI-based biomarkers to evaluate anxiety symptoms in MDD patients and their incorporation into clinical practice guidelines to establish a more objective and reliable diagnostic framework for anxious depression.

Furthermore, as a common MDD subtype, AD exhibits poorer prognosis (52). In this study, AD patients demonstrated significant structural abnormalities in brain regions including the PCUN, SPG, and ACG. As discussed, both the SPG and ACG are implicated in emotion regulation, a core mechanism in the development, maintenance, and treatment of depression and anxiety disorders, significantly influencing treatment outcomes (42, 66–68). Psychotherapy studies targeting improved emotion regulation demonstrate that enhancing this capacity significantly improves prognosis in depression and anxiety (66, 67). This may explain the poorer prognosis observed in AD patients. Moreover, abnormalities in regions like the ACG may relate to executive function deficits, potentially impacting daily functional recovery and contributing to poorer outcomes (69). This aligns with task-based fMRI findings by Bashford-Largo et al., showing that adolescents with anxiety disorders exhibit poorer task performance under emotional interference and reduced activation in the PCUN and anterior cingulate cortex (64). While consistent with existing literature, the limited scope of this study necessitates future longitudinal investigations to further elucidate the relationship between these neural correlates and disease prognosis.

Several limitations of this study should be carefully considered. Primarily, notable demographic disparities in age were observed among the AD, NAD, and HC groups. Although these variables were incorporated as covariates in our statistical analyses, the potential confounding effects cannot be entirely ruled out. Future investigations should be conducted with more balanced intergroup data to address this limitation. Secondly, while the multicenter nature of this study enhances the generalizability of our findings, it inevitably introduces data limitations, including incomplete medication records and insufficient documentation of disease progression. Importantly, these limitations preclude our ability to further investigate the potential impacts of medication details (e.g., type, dosage, duration) and disease course (e.g., frequency of episodes, changes in severity) on the study outcomes. Subsequent research should prioritize comprehensive clinical data collection, with particular attention to controlling for confounding factors such as medication use and comorbidities, to ensure more robust and reliable outcomes. Third, the lack of HAMD score information in the external validation dataset may have compromised the rigorous selection of AD patients, potentially introducing bias in the SVR model’s performance. Future studies should validate these findings using larger, more comprehensive datasets with complete clinical annotations. Fourth, although site effects were adjusted for as covariates across centers, they may still bias our findings. Future studies should validate our results in large single-center samples.

## 5 Conclusions

Our findings reveal distinct structural patterns in the brain organization of AD patients. Specifically, we identified significant GMV alterations in the right PCUN and right SPG in AD patients, along with distinct SC patterns between the right PCUN and both the right ANG and left ACG. These structural alterations may serve as neuroimaging biomarkers for AD and could potentially predict anxiety severity in patients with MDD. Collectively, these results advance our understanding of structural alterations in AD, with implications for biomarker development and pathophysiology exploration.

## Data Availability

Publicly available datasets were analyzed in this study. This data can be found here: https://www.scidb.cn/en/detail?dataSetId=cbeb3c7124bf47a6af7b3236a3aaf3a8.
