# Supplementary material for: Structural brain alterations in patients with anxious depression: evidence from the REST-meta-MDD project
Source: Front Psychiatry. 2025 Jul 25;16:1589040. doi: 10.3389/fpsyt.2025.1589040 (PMC12332750; doi:10.3389/fpsyt.2025.1589040)
Supplement: Supplementary file 1 [file SupplementaryFile1.docx]

**Supplementary Information**

**Supplementary Tables**

**Supplementary Table 1** Participating Centers' Characteristics: Demographic Data, Acquisition Parameters, and Individual Site Contributions

**Supplementary Figures**

**Supplementary Figure 1** Intergroup Comparison Results in the <25-Year-Old Cohort. **A** Brain Regions Showing Significant GMV Differences among three Groups. **B** Brain Regions Showing Significant GMV Differences Between AD and NAD Groups. **C** Brain Regions Showing Significant GMV Differences Between AD and HC Groups.

**Supplementary Figure 2** Intergroup Comparison Results in the 25–40-Year-Old Cohort. **A** Brain Regions Showing Significant GMV Differences among three Groups. **B** Brain Regions Showing Significant GMV Differences Between AD and NAD Groups. **C** Brain Regions Showing Significant GMV Differences Between AD and HC Groups.

**Supplementary Figure 3** Intergroup Comparison Results in the >40-Year-Old Cohort. **A** Brain Regions Showing Significant GMV Differences among three Groups. **B** Brain Regions Showing Significant GMV Differences Between AD and NAD Groups. **C** Brain Regions Showing Significant GMV Differences Between AD and HC Groups.

**Supplementary Table 1** Participating Centers' Characteristics: Demographic Data, Acquisition Parameters, and Individual Site Contributions

| Serial Number | Sample size | Gender (M/ F) ^a^ | Age (years) | Education (years) | Scanner | Receive (coil) | TR (ms) | TE (ms) | Flip Angle (∘) | Thickness/gap | Slice number | Time points | Voxel size | FOV |
| --- | --- | --- | --- | --- | --- | --- | --- | --- | --- | --- | --- | --- | --- | --- |
| 1 | 13 | 8/ 5 | 41.08±11.35 | 13.38±4.19 | Philips Achieva 3T | 8 channel | 2000 | 30 | 90 | 4.0mm/0 mm | 37 | 200 | 1.67 × 1.67 × 4.00 | 240 × 240 |
| 2 | 14 | 4/ 10 | 26.5±8.17 | 9.79±4.21 | GE Signa 3T | 8 channel | 2000 | 30 | 90 | 3.0mm/0mm | 35 | 200 | 3.75 × 3.75 × 3.00 | 240 × 240 |
| 3 | 11 | 6/ 5 | 30.18±10.40 | 12.27±3.64 | Siemens Tim Trio 3T | 32 channel | 2000 | 30 | 90 | 3.0mm/1.52mm | 32 | 212 | 3.75 × 3.75 × 4.52 | 240 × 240 |
| 4 | 12 | 4/ 8 | 20.50±2.24 | 13.42±1.17 | GE Signa 3T | 8 channel | 2000 | 40 | 90 | 4.0mm/0mm | 33 | 240 | 3.75 × 3.75 × 4.00 | 240 × 240 |
| 5 | 96 | 31/ 65 | 39.43±14.70 | 11.14±3.59 | Siemens Tim Trio 3T | 12 channel | 2000 | 30 | 90 | 3.0mm/1.0mm | 32 | 242 | 3.44 × 3.44 × 4.00 | 220 × 220 |
| 6 | 14 | 7/ 7 | 33.21±12.32 | 11.86±2.85 | Siemens Tim Trio 3T | 32 channel | 2000 | 30 | 90 | 3.5mm/0.7mm | 33 | 240 | 3.12 × 3.12 × 4.20 | 200 × 200 |
| 7 | 46 | 25/ 21 | 29.50±10.11 | 12.5±2.57 | Philips Gyroscan Achieva 3.0T | 32 channel | 2000 | 30 | 90 | 4.0mm/0mm | 36 | 250 | 1.67 × 1.67 × 4.00 | 240 × 240 |
| 8 | 30 | 12/ 18 | 32.17±13.10 | 13.97±4.87 | Philips Achieva 3.0T TX | 8 channal | 2000 | 30 | 90 | 4.0mm/0mm | 38 | 240 | 3.75 × 3.75 × 4.00 | 240 × 240 |
| 9 | 31 | 12/ 19 | 31.00±8.41 | 14.16±1.93 | GE Signa 1.5T | 8 channel | 2000 | 40 | 90 | 5/1mm | 24 | 160 | 3.75 × 3.75 × 6.00 | 240 × 240 |

**
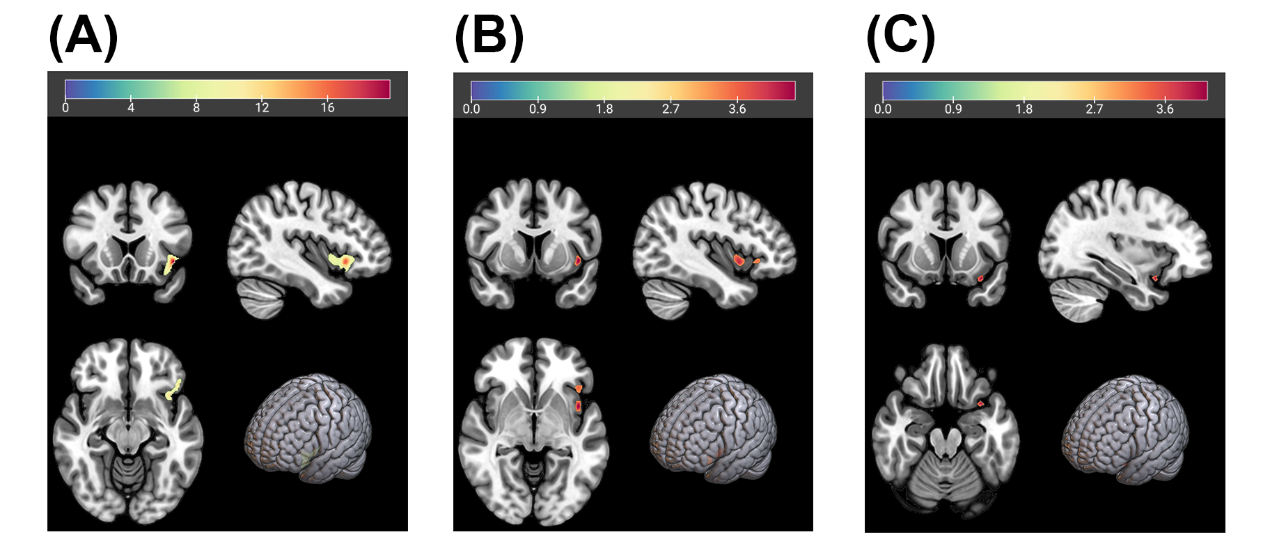
**

**Supplementary Figure 1** Intergroup Comparison Results in the <25-Year-Old Cohort. **A** Brain Regions Showing Significant GMV Differences among three Groups. **B** Brain Regions Showing Significant GMV Differences Between AD and NAD Groups. **C** Brain Regions Showing Significant GMV Differences Between AD and HC Groups.


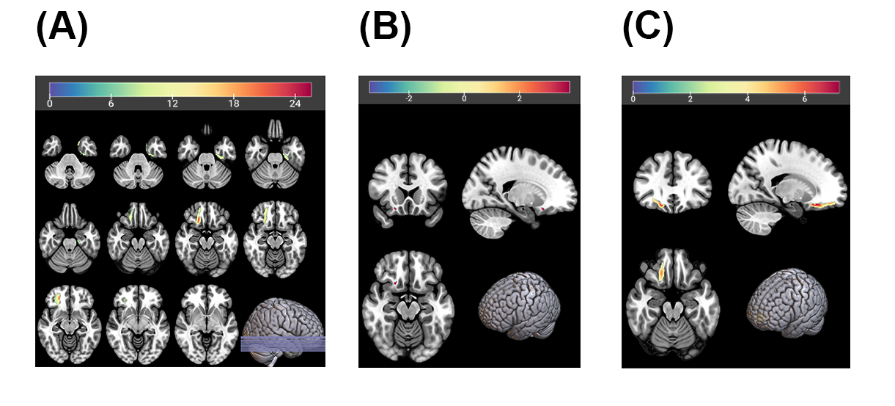


**Supplementary Figure 2** Intergroup Comparison Results in the 25–40-Year-Old Cohort. **A** Brain Regions Showing Significant GMV Differences among three Groups. **B** Brain Regions Showing Significant GMV Differences Between AD and NAD Groups. **C** Brain Regions Showing Significant GMV Differences Between AD and HC Groups.


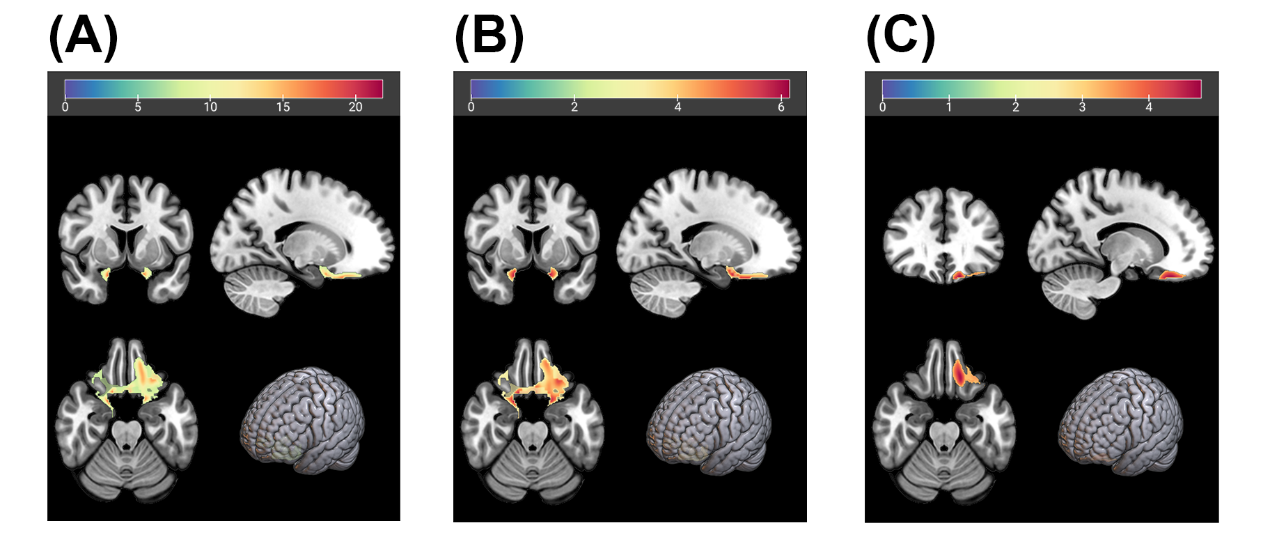


**Supplementary Figure 3** Intergroup Comparison Results in the >40-Year-Old Cohort. **A** Brain Regions Showing Significant GMV Differences among three Groups. **B** Brain Regions Showing Significant GMV Differences Between AD and NAD Groups. **C** Brain Regions Showing Significant GMV Differences Between AD and HC Groups.
